# Independent and Combined Associations of Physical Activity in Different Domains and Inflammatory Diet with Type 2 Diabetes: A Population-Based Cohort Study

**DOI:** 10.3390/nu17010047

**Published:** 2024-12-27

**Authors:** Jianfan Zhou, Shuting Yin, Litao Du, Xiangli Xue, Qiang He, Na Zhao, Si Chen, Xianliang Zhang

**Affiliations:** 1 School of Physical Education, Shandong University, 17922 Jingshi Road, Lixia District, Jinan 250061, China; 202335433@mail.sdu.edu.cn (J.Z.); 202215366@mail.sdu.edu.cn (S.Y.); 202320857@mail.sdu.edu.cn (L.D.); xiangli1277@163.com (X.X.); hq@sdu.edu.cn (Q.H.); 2 School of Sports and Health, Shandong Sport University, 10600 Century Avenue, Licheng District, Jinan 250102, China; zhaonasdtyxy@163.com; 3 School of Nursing and Rehabilitation, Cheeloo College of Medicine, Shandong University, 44 West Wenhua Road, Lixia District, Jinan 250102, China; chensi@sdu.edu.cn

**Keywords:** physical activity, diabetes, inflammatory diet, NHANES

## Abstract

Objective: This study aims to explore the independent and combined associations of physical activity (PA) in different domains and inflammatory diet with type 2 diabetes mellitus (T2DM). Methods: Data from 8736 American adults from the NHANES 2007–2016 were used. PA in different domains was assessed using the self-reported Global Physical Activity Questionnaire, and dietary inflammatory index was estimated based on 24 h dietary recalls. T2DM diagnosis was determined by a combination of self-report and laboratory data. A multivariate modified Poisson regression model was used to explore the independent and combined associations of moderate-vigorous intensity physical activity (MVPA) and inflammatory diet with T2DM. Results: PA in the Work MVPA, Recreational MVPA and Total MVPA domains was independently associated with reduced risk of T2DM, and an inflammatory diet was independently associated with elevated risk of T2DM. In the combined analysis, the combination of active and anti-inflammatory within the Work MVPA, Recreational MVPA and Total MVPA fields was associated with the greatest reduced risk of T2DM, and always associated with decreased risk of T2DM in the active group. Conclusions: Our study emphasizes that the combination of active PA and anti-inflammatory diet is closely associated with the reduced risk of T2DM, and suggests the combination of both for the prevention and treatment of T2DM.

## 1. Introduction

Type 2 Diabetes Mellitus (T2DM) is one of the most prevalent chronic diseases worldwide and is often accompanied by a range of complications, including cardiovascular disease (CVD), nerve damage, and kidney disease [1]. Despite tremendous efforts in prevention and treatment, it is predicted that T2DM will affect 1.3 billion people worldwide by 2050, resulting in approximately 11% mortality annually [2,3]. The adverse effects of T2DM and the large patient population not only seriously affect the quality of life and survival time of patients with T2DM, but also place a heavy burden on healthcare services and public health systems worldwide. Therefore, the prevention and treatment of T2DM is of paramount importance, concerning the health and well-being of hundreds of millions of people.

Regular physical activity (PA) is a well-established strategy for preventing chronic diseases and is essential for maintaining good health and preventing illnesses. The American Diabetes Association (ADA), the World Health Organization, and the American College of Sports Medicine concur that adults with diabetes should participate in 150–300 min of moderate-intensity aerobic physical activity or 75–150 min of vigorous-intensity aerobic physical activity weekly [4,5,6]. Observational studies have shown that PA is significantly associated with a reduced risk of type 2 diabetes [7,8]; also, randomized controlled trials have demonstrated that increasing PA can be effective against T2DM [9,10]. However, previous studies have primarily focused on PA during leisure time, with less attention given to the effects of PA in work, transportation, and other areas. Most scholars observe that different types of physical activity may have different health effects, and the influence of PA in different domains is controversial in T2DM. One study found that leisure-time PA (LTPA) reduced the risk of diabetes, while transportation-related PA (TPA) was negatively associated with diabetes prevalence only in men, and occupation-related PA (OPA) showed no association with diabetes among Korean adults [11]. Another study showed that LTPA, TPA, and OPA had a positive effect on reducing the risk of diabetes in American adults [12]. Thus, it is crucial to explore the effects of PA across different domains.

Much like PA, diet is one of the most modifiable daily behaviors and plays a vital role in maintaining overall health and preventing non-communicable diseases. Previous research has shown that unhealthy diets promote chronic inflammation [13], which increases the risk of obesity, cardiovascular disease (CVD), T2DM, and other non-communicable diseases [14,15]. To evaluate the inflammatory potential of the overall diet, researchers have developed the Dietary Inflammation Index (DII), which captures the intake of various parameters (foods, nutrients, and flavorings) to quantify the inflammatory potential of people’s diets on a continuum from maximally pro-inflammatory to maximally anti-inflammatory [16]. Recent studies have demonstrated that inflammatory diets, as assessed by the DII, are associated with a variety of non-communicable diseases, including T2DM [17,18,19].

PA and diet, as integral components of daily life, often have combined effects on an individual’s physical and mental health. Studies have shown that the relationship between PA and different dietary patterns is associated with depression [20], obesity [21], non-communicable diseases such as T2DM [22], all-cause mortality [23] and other health outcomes [24,25]. Furthermore, randomized controlled trials have found that PA combined with dietary and other lifestyle interventions can be effective in combating a wide range of chronic diseases [26,27,28]. Given that PA in different domains may have distinct health effects, the combined effects of PA and the DII across various domains may also differ. However, the joint association of PA across different domains and the DII with T2DM is not yet fully understood. Based on this, the present study investigated the independent and joint associations of different domains of PA (Work MVPA, Walk/bicycle MVPA, Recreational MVPA, and Total MVPA) and DII with the risk of type 2 diabetes mellitus in a population-based cohort of adults aged 18 years and older. Supplementing the evidence on the association of modifiable lifestyle factors such as PA and diet with T2DM provides new recommendations and insights for utilizing the combined effects of PA and DII to combat T2DM.

## 2. Materials and Methods

### 2.1. Study Design and Participants

Participants in this study were drawn from the National Health and Nutrition Examination Study (NHANES) 2007–2016. The NHANES is a cross-sectional survey conducted by the National Center for Health Statistics (NCHS) of the Centers for Disease Control and Prevention in the United States, designed to assess the health and nutritional status of the noninstitutionalized civilian population in the United States. NHANES uses a stratified, multistage probability sampling method, covering data on health, demographics, socioeconomic status, and diet-related aspects. A detailed description is available at https://wwwn.cdc.gov/nchs/nhanes/Default.aspx (accessed 5 October 2024). We included all adults with valid data on PA, diet, and diabetes from NHANES 2007–2016, and a total of 8736 participants were included in the final analyses after excluding participants with missing primary information, missing covariates, pregnancy, and those with T1DM (Supplementary Figure S1). NHANES was granted Ethics Review Board of the National Center for Health Statistics approval, and participants provided written informed consent.

### 2.2. Physical Activity Assessment

NHANES uses the Global Physical Activity Questionnaire to assess PA. Measures of PA correspond to three common domains: Work MVPA, Walk/bicycle MVPA, and Recreational MVPA. Work MVPA includes all work-related PA, such as paid or unpaid work, household chores, and yard work. Walk/bicycle MVPA is PA that involves traveling to and from places by walking or biking, such as to school, shopping, and work. Exercise, fitness, and other leisure activities were then categorized as Recreational MVPA. Participants reported the frequency, intensity, and duration of time spent in different domains of PA during the week. The amount of time participants devoted to moderate-intensity versus vigorous-intensity exercise in each of the three domains of PA was multiplied by the number of days in the week that they participated in both intensities to calculate the total moderate-intensity versus vigorous-intensity exercise time, and the amount of time spent in vigorous PA was doubled and added to the weekly moderate PA to obtain the weekly MVPA for each domain. This is also consistent with the intensity based on MET calculations. For example, the suggested MET scores in the NHANES manual are as follows: Moderate work-related activity is 4.0 MET, Vigorous work-related activity is 8.0 MET; Walking or bicycling for transportation is 4.0 MET; Moderate recreational physical activity is 4.0 MET, and Vigorous recreational physical activity is 8.0 MET [12,29]. In addition, a total physical activity time (Total MVPA) was calculated by summing the minutes in these three domains. According to the World Health Organization’s 2020 guidelines for physical activity and sedentary behavior [4], one can further categorize MVPA from different domains and total MVPA into inactive (<150 min) and active (≥150 min).

### 2.3. Assessment of Inflammatory Diets

NHANES used 24 h dietary recall interviews (24HR) to assess dietary information. Trained interviewers interviewed each participant to gather information about the types and amounts of foods and beverages consumed by the participant in the past 24 h and to estimate the energy and nutrients consumed from these foods and beverages. The DII judges the inflammatory potential of an individual’s diet based on 45 pro- and anti-inflammatory food parameters [16]. In NHANES, 28 food parameters were used for DII calculations, mainly including vitamins A/B6/B12/C/D/E, β-carotene, iron, magnesium, zinc, selenium, caffeine, alcohol, *n*-3 fatty acids, *n*-6 fatty acids, protein, carbohydrates, cholesterol, fiber, folic acid, niacin, total fat, riboflavin, saturated fat, monounsaturated fat, polyunsaturated fat and thiamine and energy. Nutritional and final energy-adjusted DII per 1000 calories consumed was calculated using the energy density method. DII < 0 and ≥0 represent anti-inflammatory and pro-inflammatory diets, respectively [30].

### 2.4. Diagnosis of Type 2 Diabetes Mellitus

Participants who met any of the following criteria were considered diabetic: (1) participants with a self-reported diagnosis of diabetes mellitus; (2) use of insulin or oral hypoglycemic medication; (3) fasting glucose ≥ 7.0 mmol/L (126 mg/dL); and (4) glycated hemoglobin A1c (HbA1c) levels ≥ 6.5% (47.5 mmol/mol). In addition, patients with probable type 1 diabetes mellitus (T1DM) were excluded, with T1D defined as patients receiving only insulin therapy and <20 years of age [31,32].

### 2.5. Covariates

We selected covariates that could theoretically be associated with PA, inflammatory diet, and T2DM. These included age (<60 years, ≥60 years), sex, race/ethnicity (Mexican American, Other Hispanic, Non-Hispanic White, Non-Hispanic Black, Other), educational attainment (Less than 9th grade, 9th–11th grade, High school graduate, Some college or AA degree, College graduate or above), Poverty Income Ratio (PIR) (≤1.3, 1.3 < PIR ≤ 3.5, >3.5), marital status (Married, Widowed, Divorced, Separated, Never married, Living with partner), body mass index (BMI) (<25, 25 ≤ BMI < 30, ≥30), smoking status (yes, no), drinking status (yes, no), hypertension (yes, no), CVD (yes, no), stroke (yes, no), cancer (yes, no).

### 2.6. Statistical Analysis

All analyses considered the complex, multi-stage and probabilistic clustering design of NHANES. Categorical variables were expressed as numbers (percentages) and groups were compared using chi-square tests. Multivariate modified Poisson regression models were used to determine associations between PA and type 2 diabetes, inflammatory diet and type 2 diabetes, and the joint association of PA and inflammatory diet with type 2 diabetes. Covariates were adjusted in multiple models: model 1 adjusted for age, sex, and race/ethnicity; model 2 adjusted for BMI, education, PIR, marital status, smoking status, and drinking status based on model 1; and model 3 additionally adjusted for hypertension, stroke, CVD, cancer, and PA/inflammatory diet based on model 2. Three-dimensional histograms of sample-weighted prevalence were plotted using Origin 2024 based on the joint association of different PA types with inflammatory diet. Subgroup analyses were performed including age (<60, ≥60), sex (male, female), BMI (<25, ≥25 and <30, ≥30), smoking status (smoker, nonsmoker), and drinking status (drinker, nondrinker). Likelihood ratio tests were used to obtain *p*-values for the interaction between different combinations of PA and diet and for each subgroup of factors by comparing the likelihood values of the interaction model (including the multiplicative interaction term) with those of the main model (excluding the multiplicative interaction term). All analyses were performed using StataMP 17 (StataCorp, College Station, TX, USA) and R V4.4.1 (R Foundation for Statistical Computing, Vienna, Austria) software, and a two-sided *p* value < 0.05 was considered statistically significant.

## 3. Results

### 3.1. Basic Characteristics of the Participants

A total of 8736 U.S. adults were included in this study, with 51.57% females, 65.28% adults < 60 years old, 25.77% anti-inflammatory diets, and 1626 patients with T2DM, with a prevalence of approximately 18.61%. There were different proportions in the number of active participants between different areas of PA, Work MVPA: 34.24%, Walk/bicycle MVPA: 13.51%, Recreational MVPA: 33.28% and Total MVPA: 60.52%. Statistically significant differences (*p* < 0.001) were found between the normal group and T2DM patients in terms of age, gender, BMI, race/ethnicity, education, PIR, marital status, smoking status, alcohol use, hypertension, stroke, CVD, and cancer, as shown in Table 1.

### 3.2. Independent Association of PA and Inflammatory Diets with Type 2 Diabetes Mellitus

Table 2 shows the results of multivariate logistic regression between different domains of PA and inflammatory diet and T2DM. In Model 1, different domains of MVPA and total MVPA were positively and significantly associated with T2DM compared to inactive participants (Recreational MVPA: RR = 0.57, 95% CI: 0.47, 0.70; Work MVPA: RR = 0.80, 95% CI: 0.68, 0.94; Walk/bicycle MVPA: RR = 0.69, 95% CI: 0.56, 0.84; Total MVPA: RR = 0.59, 95% CI: 0.51, 0.68). In the full adjusted model, the associations of Recreational MVPA, Work MVPA and Total MVPA with T2DM remained; however, the association of Walk/bicycle MVPA with T2DM was no longer statistically significant (RR = 0.87, 95% CI: 0.71, 1.06). In the dietary model, the pro-inflammatory diet had a negative significant association with T2DM compared to participants on the anti-inflammatory diet in the fully adjusted model (RR = 1.17, 95% CI: 1.01, 1.36).

### 3.3. Joint Association of PA with Inflammatory Diet and Type 2 Diabetes Mellitus

**Figure 1:**
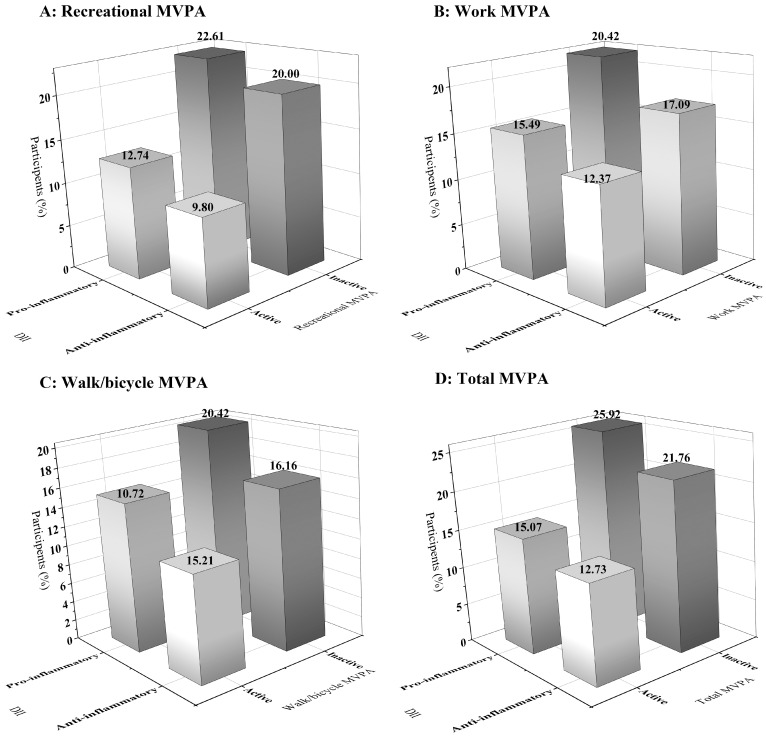
Sample-weighted prevalence of PA in combination with inflammatory diet and T2DM in different domains.

Figure 1 illustrates the weighted prevalence of T2DM in the combination of PA and inflammatory diet in different domains. Among all domains of PA, the prevalence of T2DM was lowest in the combination of active and anti-inflammatory diets, highest in the combination of inactive and inflammatory diets, and as high as 25.92% in the combination of inactive and inflammatory diets in Total MVPA. It is worth noting that changing either the inactive PA or the inflammatory diet caused a decrease in the prevalence of T2DM, and similar results were found in all physical activity domains.

Table 3 demonstrates the combined effects of different domains of PA and inflammatory diet with T2DM. Among different domains, MVPA and total MVPA, compared with Inactive and Pro-inflammatory (Recreational MVPA: RR = 0.62, 95% CI: 0.50, 0.77; Work MVPA: RR = 0.81, 95% CI: 0.67, 0.97; Walk/bicycle MVPA: RR = 0.70, 95% CI: 0.57, 0.85; Total MVPA: RR = 0.61, 95% CI: 0.52, 0.71) and Active and Anti-inflammatory (Recreational MVPA: RR = 0.42, 95% CI: 0.31, 0.58; Work MVPA: RR = 0.55, 95% CI: 0.41, 0.72; Walk/bicycle MVPA: RR = 0.47, 95% CI: 0.31, 0.73; Total MVPA: RR = 0.45, 95% CI: 0.37, 0.55) were all positively and significantly associated with T2DM. In the fully adjusted model, except for Walk/bicycle MVPA, which was not associated with T2DM (RR = 0.75, 95% CI: 0.48, 1.18), the associations remained (Recreational MVPA: RR = 0.66, 95% CI: 0.49, 0.90; Work MVPA: RR = 0.66, 95% CI: 0.49, 0.88; Total MVPA: RR = 0.68, 95% CI: 0.55, 0.83). In addition, we observed that in Recreational MVPA (RR: 0.81 vs. 0.66), Work MVPA (RR: 0.784 vs. 0.66), and Total MVPA (RR: 0.77 vs. 0.68), the RRs of Active and Anti-inflammatory participants were reduced compared to Active and Pro-inflammatory participants.

### 3.4. Subgroup Analyses

Supplementary Figures S2–S6 show the results of the subgroup analysis. Among participants aged 60 years and older, the active and anti-inflammatory combinations of Recreational MVPA domains were positively associated with T2DM (Supplementary Figure S2). Among men, active and anti-inflammatory combinations of Recreational MVPA, Work MVPA, and Total MVPA were positively associated with T2DM (Supplementary Figure S3). Among non-smoking participants, the active and anti-inflammatory combinations of Work MVPA and Total MVPA were positively associated with T2DM (Supplementary Figure S4). Among participants who consumed alcohol, the active and anti-inflammatory combinations of Recreational MVPA and Work MVPA were positively associated with T2DM (Supplementary Figure S5). Among participants with a BMI ≥ 30, inactivity and pro-inflammatory alterations in any of the Recreational MVPA, Work MVPA, and Total MVPA domains were positively associated with T2DM (Supplementary Figure S6).

## 4. Discussion

This study investigated the independent and joint associations of MVPA and inflammatory diet with T2DM across domains based on nationally representative NHANES 2007–2016 data. Our results showed that anti-inflammatory diet, Recreational MVPA, Work MVPA, and Total MVPA were all significantly and independently negatively associated with T2DM. The combination of Active and anti-inflammatory diet had a more positive and significant effect on T2DM. This suggests to us that performing adequate MVPA along with an anti-inflammatory diet may be potentially important for the prevention of T2DM.

There is a general consensus regarding the relationship between PA and T2DM. Numerous studies have demonstrated a positive effect of PA on T2DM [2,8,33,34,35,36], and several guidelines have suggested that patients with T2DM should increase their daily PA levels. Our findings are largely in line with previous studies that have shown a correlation between increased levels of PA and a reduced risk of developing T2DM. However, while earlier studies have primarily focused on recreational MVPA, our study expands on this by exploring the associations of MVPA in transportation and work-related domains. Our research indicated that not only were Recreational MVPA levels significantly correlated with T2DM, but Work MVPA and Total MVPA were also notably linked to the condition. These findings are consistent with previous studies [12]. Notably, Walk/bicycle MVPA did not seem to be significantly associated with T2DM in our study. Previous studies have also found no association with depression and coronary heart disease [37,38]. This discrepancy may be influenced by factors such as race/ethnicity and gender, as some studies suggest that walking and cycling are more strongly associated with a reduced risk of T2DM in non-Latino whites compared to Latinos [12], and that the effect of Walk/bicycle MVPA on the development of T2DM in women is more significant [39]. Nonetheless, it is undeniable that PA has a positive impact on T2DM prevention. Therefore, we urge individuals, particularly those with T2DM, to increase their MVPA across different domains to support the management and prevention of the condition.

In terms of inflammatory diet, our study found that a pro-inflammatory diet was associated with a higher risk of T2DM, which is consistent with previous findings [17,19]. A pro-inflammatory diet may affect T2DM through several mechanisms. First, it increases circulating levels of pro-inflammatory cytokines, such as IL-6 and C-reactive protein, which promote chronic systemic inflammation—a key pathophysiological feature and pathogenic mechanism of T2DM [9,40,41]. Furthermore, it has been found that pro-inflammatory diets are associated with the development of insulin resistance [42,43], a major risk factor for T2DM. This suggests that pro-inflammatory diets may raise the risk of T2DM by exacerbating insulin resistance. Finally, inflammatory diets may worsen the risk of T2DM by disrupting glucose metabolic homeostasis. Research has shown that pro-inflammatory diets are significantly associated with elevated glucose metabolic indices, including fasting plasma glucose (FPG), fasting serum insulin (FSI), and the homeostasis model assessment of insulin resistance (HOMA-IR) [43].

Previous studies have primarily focused on the independent associations of PA and inflammatory diets with T2DM, often overlooking their combined effects, and we extend the evidence. To our knowledge, this is the first study to investigate the combined effect of PA and inflammatory diet on T2DM based on a nationally representative sample. The joint analysis allowed us to explore the unique and combined contribution of each factor to T2DM, providing a more comprehensive guide to preventing T2DM. Our joint analysis showed that the active PA group was always negatively associated with T2DM in Recreational MVPA, Work MVPA, and Total MVPA, regardless of inflammatory diet. This suggests that PA is strongly associated with a lower risk of T2DM, underscoring its significant potential in combating the disease. A similar study investigating the combined effects of PA and the DII on depression found comparable results, indicating that inactive physical activity carries a higher risk of depressive symptoms than a pro-inflammatory diet alone [44]. Furthermore, our results suggest that the combination of physical activity and an anti-inflammatory diet has the greatest effect on reducing T2DM risk. In fact, previous studies have shown that the combination of more active PA and an anti-inflammatory diet offers substantial protective effects against conditions such as stroke [45], cognitive function [46], obesity [21], mortality and depression [44]. This supports the idea that synergistic changes, combining both PA and diet, may be more effective than modifying either factor in isolation when it comes to reducing T2DM risk and improving overall health. This emphasizes the importance of integrating PA with dietary modifications, two modifiable lifestyle factors that may offer substantial benefits. Several randomized controlled trials have demonstrated this approach’s effectiveness: for instance, a three-year lifestyle intervention in Finland, combining PA with diet, significantly improved glycemic and lipid markers and reduced diabetes risk among participants [47]. Similar results have been observed in obese populations [48], and intensive lifestyle interventions have been shown to delay the progression of early-stage T2DM in younger patients, reduce body weight, and improve cardiometabolic health [49]. Therefore, we recommend that individuals adopt a more aggressive approach to PA combined with an anti-inflammatory diet in their daily lives, as this combination holds great potential for the prevention and management of T2DM.

Our study has several strengths. First, we are the first to investigate the combined effects of PA and an inflammatory diet on T2DM across various domains, offering new insights into the prevention of T2DM through modifiable lifestyle factors. Second, we employed sample weighting and multivariate adjustment in all regression models to enhance the reliability and robustness of our findings. Additionally, we categorized different domains of PA and examined the interaction between these domains and an inflammatory diet in relation to T2DM. However, there are some limitations to our study. First, both PA and dietary data were self-reported, which may be subject to recall bias. Despite this, self-reported PA allowed us to categorize various PA domains, a process that would not be feasible with objectively measured PA. Regarding the inflammatory diet, we excluded individuals who participated in only one 24 h dietary recall to improve the validity of our results. Second, our study was cross-sectional, which limits our ability to assess changes in individual dietary or activity patterns over time. As a result, the data represent associations rather than causal relationships. Therefore, further prospective studies or randomized controlled trials are needed for more in-depth analyses.

## 5. Conclusions

In conclusion, active Recreational MVPA, Work MVPA, Total MVPA, and anti-inflammatory diets were each associated with a reduced risk of T2DM. In the joint analysis, active PA was consistently linked to a lower risk of T2DM, regardless of dietary habits. This finding suggests that individuals should aim to increase physical activity, particularly recreational MVPA. For those who find it challenging to increase recreational MVPA, engaging in Work MVPA—such as brisk walking, housework, and gardening—can still yield significant health benefits in terms of T2DM risk reduction. Notably, the combination of active PA with an anti-inflammatory diet was associated with the greatest reduction in T2DM risk. This highlights the potential for synergistic effects between physical activity and a healthy diet in combating T2DM. Therefore, while prioritizing physical activity is important, the role of diet in maintaining health should not be overlooked.

## Figures and Tables

**Table 1:** Basic characteristics of participants.

Variables	Total N = 8736	Normal N = 7110	Diabetes N = 1626	*p*-Value
Age, years				<0.001
<60	5703 (65.28)	5035 (70.82)	668 (41.08)	
≥60	3033 (34.72)	2075 (29.18)	958 (58.92)	
Gender				<0.001
Male	4231 (48.43)	3377 (47.50)	854 (52.52)	
Female	4505 (51.57)	3733 (52.50)	772 (47.48)	
BMI, kg/m^2^				<0.001
<25	2492 (28.53)	2286 (32.15)	206 (12.67)	
25 ≤ BMI < 30	2911 (33.32)	2462 (34.63)	449 (27.61)	
≥30	3333 (38.15)	2362 (33.22)	971 (59.72)	
Race				<0.001
Mexican American	1248 (14.29)	982 (13.81)	266 (16.36)	
Other Hispanic	868 (9.94)	693 (9.75)	175 (10.76)	
Non-Hispanic White	4157 (47.58)	3486 (49.03)	671 (41.27)	
Non-Hispanic Black	1657 (18.97)	1253 (17.62)	404 (24.85)	
Other	806 (9.23)	696 (9.79)	110 (6.77)	
Education				<0.001
Less than 9th grade	776 (8.88)	536 (7.54)	240 (14.76)	
9–11th grade	1210 (13.85)	931 (13.09)	279 (17.16)	
High school graduate	1967 (22.52)	1562 (21.97)	405 (24.91)	
Some college or AA degree	2559 (29.29)	2129 (29.94)	430 (26.45)	
College graduate or above	2224 (25.46)	1952 (27.45)	272 (16.73)	
PIR				<0.001
≤1.3	2729 (31.24)	2184 (30.72)	545 (33.52)	
1.3 < PIR ≤ 3.5	3296 (37.73)	2619 (36.84)	677 (41.64)	
>3.5	2711 (31.03)	2307 (32.45)	404 (24.85)	
Marital status				<0.001
Married	4629 (52.99)	3704 (52.10)	925 (56.89)	
Widowed	691 (7.91)	480 (6.75)	211 (12.98)	
Divorced	955 (10.93)	753 (10.59)	202 (12.42)	
Separated	259 (2.96)	205 (2.88)	54 (3.32)	
Never married	1537 (17.59)	1377 (19.37)	160 (9.84)	
Living with partner	665 (7.61)	591 (8.31)	74 (4.55)	
Smoking status	3921 (44.88)	3110 (43.74)	811 (49.88)	<0.001
Drinking status	6342 (72.60)	5281 (74.28)	1061 (65.25)	<0.001
Hypertension	3762 (43.06)	2593 (36.47)	1169 (71.89)	<0.001
Stroke	332 (3.80)	206 (2.90)	126 (7.75)	<0.001
CVD	749 (8.57)	426 (5.99)	323 (19.86)	<0.001
Cancer	849 (9.72)	605 (8.51)	244 (15.01)	<0.001
Work MVPA				<0.001
Inactive	5745 (65.76)	4556 (64.08)	1189 (73.12)	
Active	2991 (34.24)	2554 (35.92)	437 (26.88)	
Walk/bicycle MVPA				<0.001
Inactive	7556 (86.49)	6094 (85.71)	1462 (89.91)	
Active	1180 (13.51)	1016 (14.29)	164 (10.09)	
Recreational MVPA				<0.001
Inactive	5829 (66.72)	4543 (63.90)	1286 (79.09)	
Active	2907 (33.28)	2567 (36.10)	340 (20.91)	
Total MVPA				<0.001
Inactive	3449 (39.48)	2582 (36.32)	867 (53.32)	
Active	5287 (60.52)	4528 (63.68)	759 (46.68)	
Inflammatory diet				<0.001
Anti-inflammatory	2251 (25.77)	1906 (26.81)	345 (21.22)	
Pro-inflammatory	6485 (74.23)	5204 (73.19)	1281 (78.78)	

Note: BMI, body mass index; PIR, poverty index ratio; MVPA, moderate-to-vigorous physical activity; CVD, cardiovascular disease. Categorical variables were expressed as numbers (percentages) and groups were compared using the chi-square test.

**Table 2:** Association between physical activity and pro-inflammatory diet with diabetes (weekly moderate-to-vigorous physical activity <150 min and pro-inflammatory diet as reference).

	Model 1		Model 2		Model 3	
	RR (95% CI)	*p*-Value	RR (95% CI)	*p*-Value	RR (95% CI)	*p*-Value
Recreational MVPA	0.57 (0.47, 0.70)	**<0.001**	0.76 (0.64, 0.90)	**0.002**	0.80 (0.68, 0.94)	**0.008**
Work MVPA	0.80 (0.68, 0.94)	**0.006**	0.79 (0.68, 0.93)	**0.004**	0.82 (0.70, 0.96)	**0.015**
Walk/bicycle MVPA	0.69 (0.56, 0.84)	**<0.001**	0.84 (0.68, 1.03)	0.087	0.87 (0.71, 1.06)	0.163
Total MVPA	0.59 (0.51, 0.68)	**<0.001**	0.72 (0.63, 0.83)	**<0.001**	0.78 (0.68, 0.89)	**<0.001**
Pro-inflammatory diet	1.44 (1.26, 1.66)	**<0.001**	1.19 (1.03, 1.39)	**0.020**	1.17 (1.01, 1.36)	**0.039**

Note: RR, relative risk; CI, confident interval. Model 1: adjusted for age, sex, and race/ethnicity; Model 2: additionally adjusted for education, PIR, marital status, BMI, smoking status, drinking status; Model 3: additionally adjusted for hypertension, stroke, CVD, cancer, and inflammatory diet/total MVPA based on Model 2. *p*-values less than 0.05 are highlighted in bold to indicate statistical significance.

**Table 3:** Association of Physical Activity and Inflammatory Diet Joint with Diabetes (Inactive & Pro-inflammatory as reference).

	Model 1		Model 2		Model 3	
	RR (95% CI)	*p*-Value	RR (95% CI)	*p*-Value	RR (95% CI)	*p*-Value
Recreational MVPA						
Inactive and Pro-inflammatory	Ref		Ref		Ref	
Inactive and Anti-inflammatory	0.79 (0.65, 0.95)	**0.012**	0.87 (0.72, 1.05)	0.147	0.87 (0.72, 1.05)	0.140
Active and Pro-inflammatory	0.62 (0.50, 0.77)	**<0.001**	0.78 (0.64, 0.96)	**0.018**	0.81 (0.67, 0.99)	**0.045**
Active and Anti-inflammatory	0.42 (0.31, 0.58)	**<0.001**	0.64 (0.47, 0.87)	**0.005**	0.66 (0.49, 0.90)	**0.008**
Work MVPA						
Inactive and Pro-inflammatory	Ref		Ref		Ref	
Inactive and Anti-inflammatory	0.70 (0.58, 0.84)	**<0.001**	0.86 (0.71, 1.04)	0.113	0.87 (0.72, 1.05)	0.137
Active and Pro-inflammatory	0.81 (0.67, 0.97)	**0.021**	0.81 (0.67, 0.97)	**0.023**	0.84 (0.70, 1.01)	0.069
Active and Anti-inflammatory	0.55 (0.41, 0.72)	**<0.001**	0.65 (0.48, 0.87)	**0.005**	0.66 (0.49, 0.88)	**0.006**
Walk/bicycle MVPA						
Inactive and Pro-inflammatory	Ref		Ref		Ref	
Inactive and Anti-inflammatory	0.70 (0.60, 0.82)	**<0.001**	0.83 (0.70, 0.98)	**0.033**	0.83 (0.70, 0.98)	**0.031**
Active and Pro-inflammatory	0.70 (0.57, 0.85)	**<0.001**	0.82 (0.67, 1)	0.058	0.86 (0.70, 1.04)	0.121
Active and Anti-inflammatory	0.47 (0.31, 0.73)	**0.001**	0.72 (0.45, 1.17)	0.186	0.75 (0.48, 1.18)	0.211
Total MVPA						
Inactive and Pro-inflammatory	Ref		Ref		Ref	
Inactive and Anti-inflammatory	0.72 (0.57, 0.93)	**0.011**	0.80 (0.62, 1.02)	0.066	0.79 (0.61, 1.01)	0.060
Active and Pro-inflammatory	0.61 (0.52, 0.71)	**<0.001**	0.71 (0.61, 0.84)	**<0.001**	0.77 (0.65, 0.87)	**0.001**
Active and Anti-inflammatory	0.45 (0.37, 0.55)	**<0.001**	0.64 (0.52, 0.79)	**<0.001**	0.68 (0.55, 0.83)	**<0.001**

Note: RR, relative risk, CI, confident interval. Model 1: adjusted for age, sex, and race/ethnicity; Model 2: additionally adjusted for education, PIR, marital status, BMI, smoking status, drinking status; Model 3: additionally adjusted for hypertension, stroke, CVD, cancer based on Model 2. *p*-values less than 0.05 are highlighted in bold to indicate statistical significance.

## Data Availability

All data generated and analyzed during this study are derived from the National Health and Nutrition and Examination Survey 2007–2016, which are available to the public (https://www.cdc.gov/nchs/nhanes (accessed 5 October 2024)). Codes are available from the authors upon reasonable request from the reader.

## Supplementary Materials

The following supporting information can be downloaded at: https://www.mdpi.com/article/10.3390/nu17010047/s1, Figure S1: Participant flow chart; Figure S2: Subgroup and interaction analyses of the association between different domains of PA with combinations of inflammatory diet and T2DM based on age. Figure S3: Subgroup and interaction analyses of the association between different domains of PA with combinations of inflammatory diet and T2DM based on gender. Figure S4: Subgroup and interaction analyses of associations between different domains of PA based on smoking status in combination with inflammatory diet and T2DM. Figure S5: Subgroup and interaction analyses of the association between different domains of PA based on drinking status in combination with inflammatory diet and T2DM. Figure S6: Subgroup and interaction analyses of the association between different domains of PA based on BMI in combination with inflammatory diet and T2DM. Figure S7: Forest plot of the relationship between physical activity and pro-inflammatory diet with diabetes based on Model 3.
