# Supplementary material for: Independent and Combined Associations of Physical Activity in Different Domains and Inflammatory Diet with Type 2 Diabetes: A Population-Based Cohort Study
Source: Nutrients. 2024 Dec 27;17(1):47. doi: 10.3390/nu17010047 (PMC11723060; doi:10.3390/nu17010047)
Supplement: Supplementary file 1 [file nutrients-17-00047-s001.zip › nutrients-3372972-supplementary.pdf]

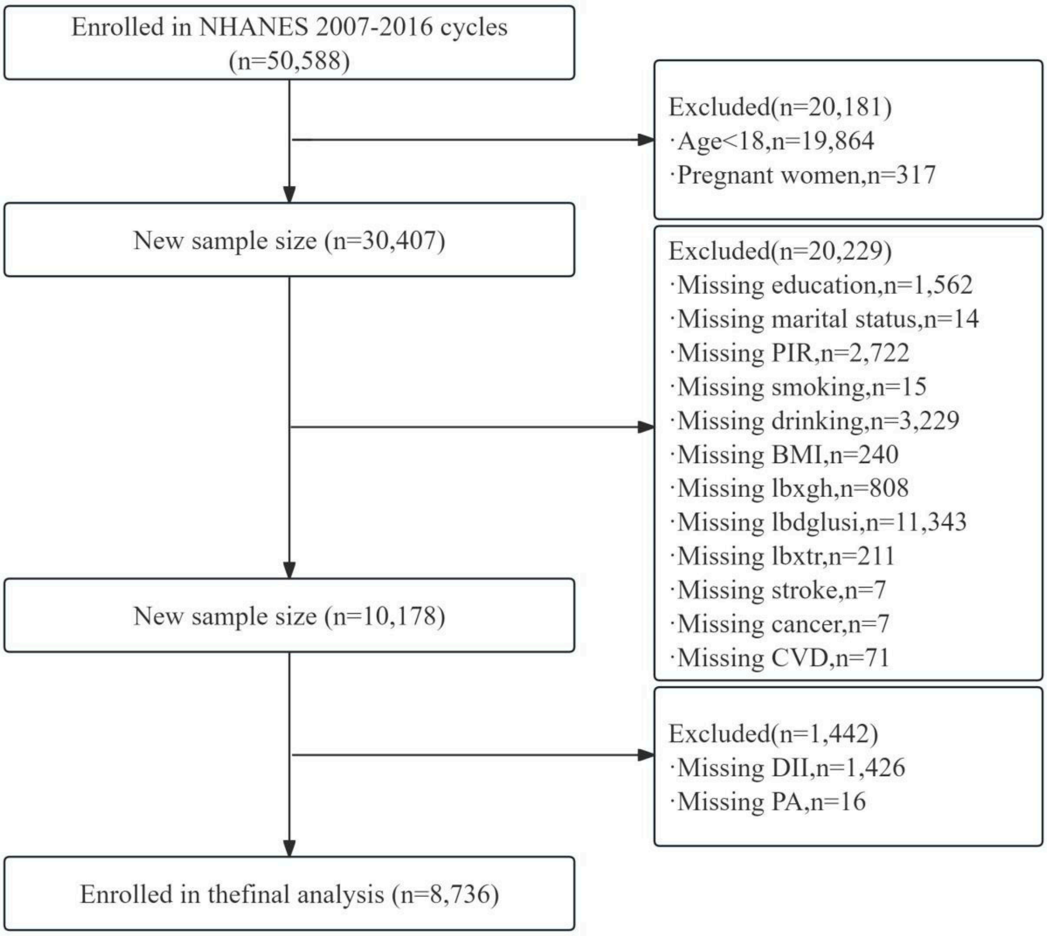

Figure S1. Participant flow chart

Variables	RR(95%CI)	P-Value for interaction
Age		<0.01
<60		
Recreational MVPA		
Inactive & Pro-inflammatory	ref.	
Inactive & Anti-inflammatory	0.90(0.67,1.22)	
Active & Pro-inflammatory	0.74(0.55,0.99)	
Active & Anti-inflammatory	0.72(0.47,1.10)	
Work MVPA		
Inactive & Pro-inflammatory	ref.	
Inactive & Anti-inflammatory	0.89(0.65,1.20)	
Active & Pro-inflammatory	0.70(0.55,0.90)	
Active & Anti-inflammatory	0.68(0.46,1.01)	
Walk/bicycle MVPA		
Inactive & Pro-inflammatory	ref.	
Inactive & Anti-inflammatory	0.90(0.69,1.17)	
Active & Pro-inflammatory	0.69(0.50,0.95)	
Active & Anti-inflammatory	0.61(0.30,1.25)	
Total MVPA		
Inactive & Pro-inflammatory	ref.	
Inactive & Anti-inflammatory	0.74(0.52,1.05)	
Active & Pro-inflammatory	0.60(0.48,0.75)	
Active & Anti-inflammatory	0.64(0.47,0.86)	
>=60		
Recreational MVPA		
Inactive & Pro-inflammatory	ref.	
Inactive & Anti-inflammatory	0.85(0.66,1.08)	
Active & Pro-inflammatory	0.93(0.70,1.23)	
Active & Anti-inflammatory	0.58(0.38,0.89)	
Work MVPA		
Inactive & Pro-inflammatory	ref.	
Inactive & Anti-inflammatory	0.87(0.66,1.23)	
Active & Pro-inflammatory	0.99(0.78,1.26)	
Active & Anti-inflammatory	0.55(0.38,0.81)	
Walk/bicycle MVPA		
Inactive & Pro-inflammatory	ref.	
Inactive & Anti-inflammatory	0.77(0.61,0.97)	
Active & Pro-inflammatory	1.11(0.86,1.42)	
Active & Anti-inflammatory	0.88(0.44,1.74)	
Total MVPA		
Inactive & Pro-inflammatory	ref.	
Inactive & Anti-inflammatory	0.86(0.61,1.20)	
Active & Pro-inflammatory	0.93(0.78,1.12)	
Active & Anti-inflammatory	0.93(0.78,1.12)	

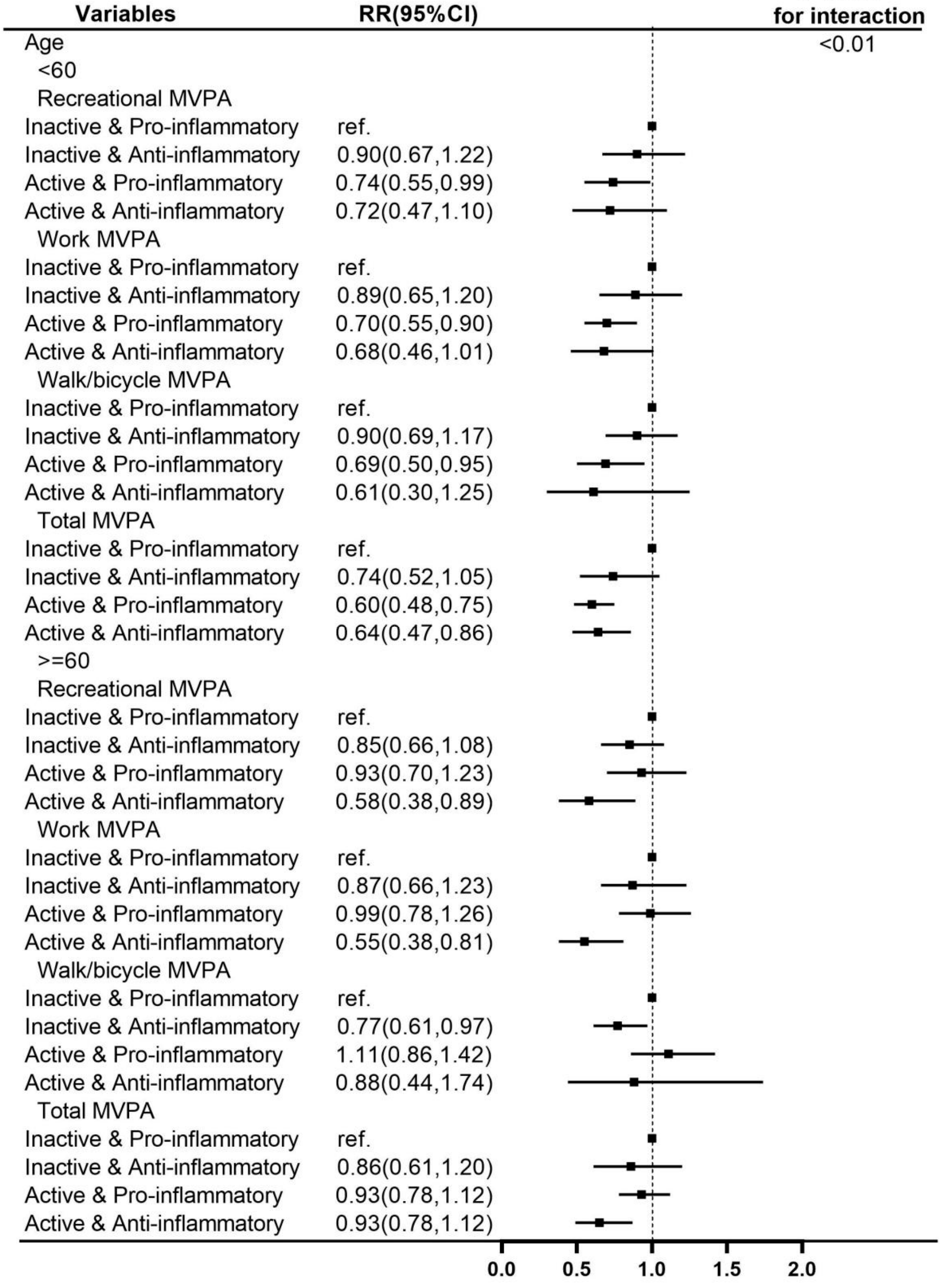

Figure S2. Subgroup and interaction analyses of the association between different domains of PA with combinations of inflammatory diet and T2DM based on age.

Variables	RR(95%CI)	P-Value for interaction
Gender		<0.01
Male		
Recreational MVPA		
Inactive & Pro-inflammatory	ref.	
Inactive & Anti-inflammatory	0.93(0.74,1.17)	
Active & Pro-inflammatory	0.84(0.61,1.15)	
Active & Anti-inflammatory	0.68(0.48,0.95)	
Work MVPA		
Inactive & Pro-inflammatory	ref.	
Inactive & Anti-inflammatory	0.91(0.73,1.14)	
Active & Pro-inflammatory	0.82(0.65,1.05)	
Active & Anti-inflammatory	0.64(0.45,0.92)	
Walk/bicycle MVPA		
Inactive & Pro-inflammatory	ref.	
Inactive & Anti-inflammatory	0.88(0.71,1.08)	
Active & Pro-inflammatory	0.82(0.67,1.02)	
Active & Anti-inflammatory	0.63(0.35,1.12)	
Total MVPA		
Inactive & Pro-inflammatory	ref.	
Inactive & Anti-inflammatory	0.76(0.58,1.00)	
Active & Pro-inflammatory	0.64(0.51,0.81)	
Active & Anti-inflammatory	0.63(0.49,0.81)	
Female		
Recreational MVPA		
Inactive & Pro-inflammatory	ref.	
Inactive & Anti-inflammatory	0.79(0.57,1.10)	
Active & Pro-inflammatory	0.83(0.62,1.12)	
Active & Anti-inflammatory	0.64(0.38,1.10)	
Work MVPA		
Inactive & Pro-inflammatory	ref.	
Inactive & Anti-inflammatory	0.77(0.56,1.05)	
Active & Pro-inflammatory	0.88(0.68,1.15)	
Active & Anti-inflammatory	0.71(0.42,1.22)	
Walk/bicycle MVPA		
Inactive & Pro-inflammatory	ref.	
Inactive & Anti-inflammatory	0.74(0.56,0.99)	
Active & Pro-inflammatory	0.95(0.66,1.37)	
Active & Anti-inflammatory	0.97(0.52,1.79)	
Total MVPA		
Inactive & Pro-inflammatory	ref.	
Inactive & Anti-inflammatory	0.76(0.50,1.17)	
Active & Pro-inflammatory	0.91(0.72,1.15)	
Active & Anti-inflammatory	0.72(0.49,1.06)	

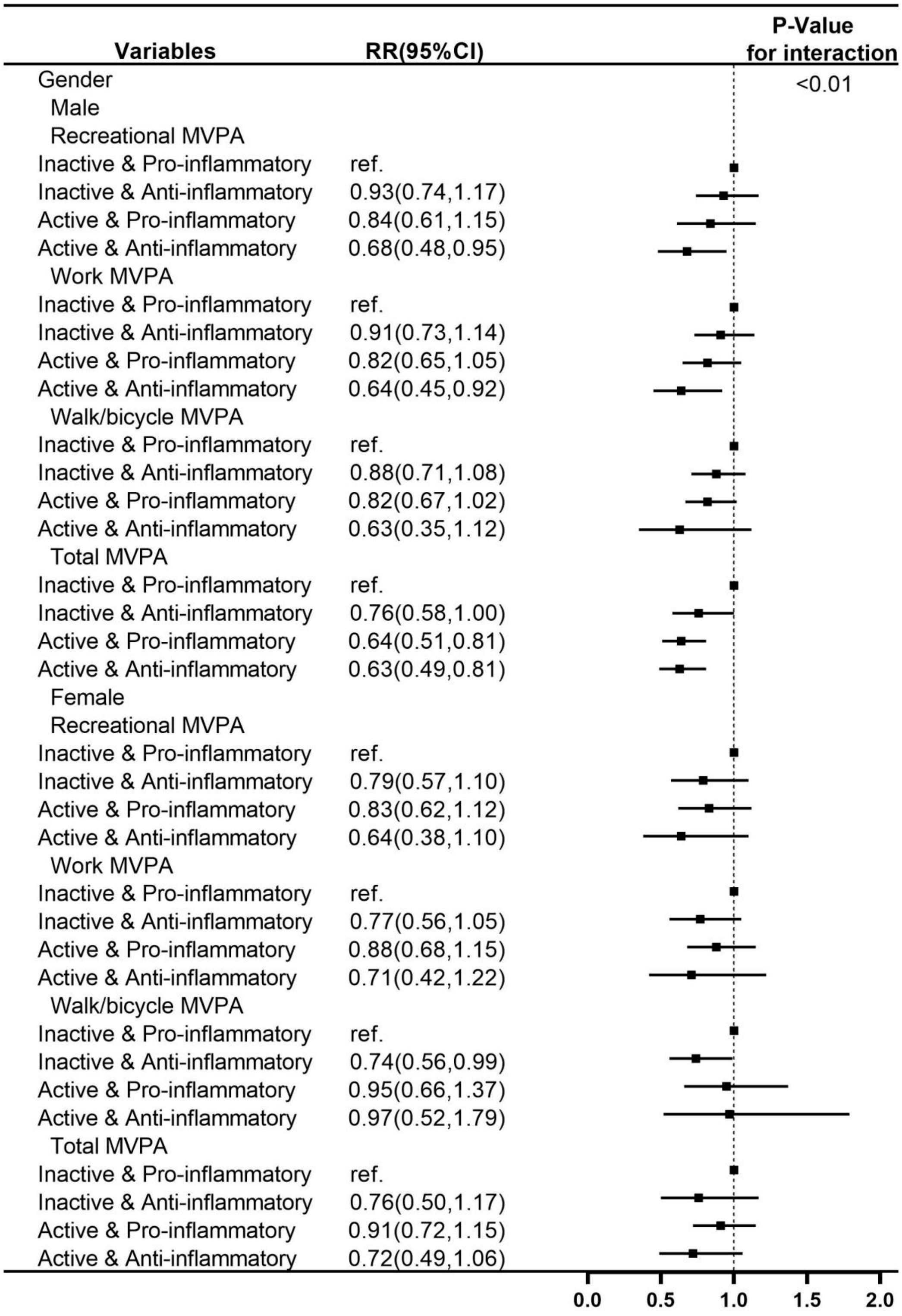

Figure S3. Subgroup and interaction analyses of the association between different domains of PA with combinations of inflammatory diet and T2DM based on gender.

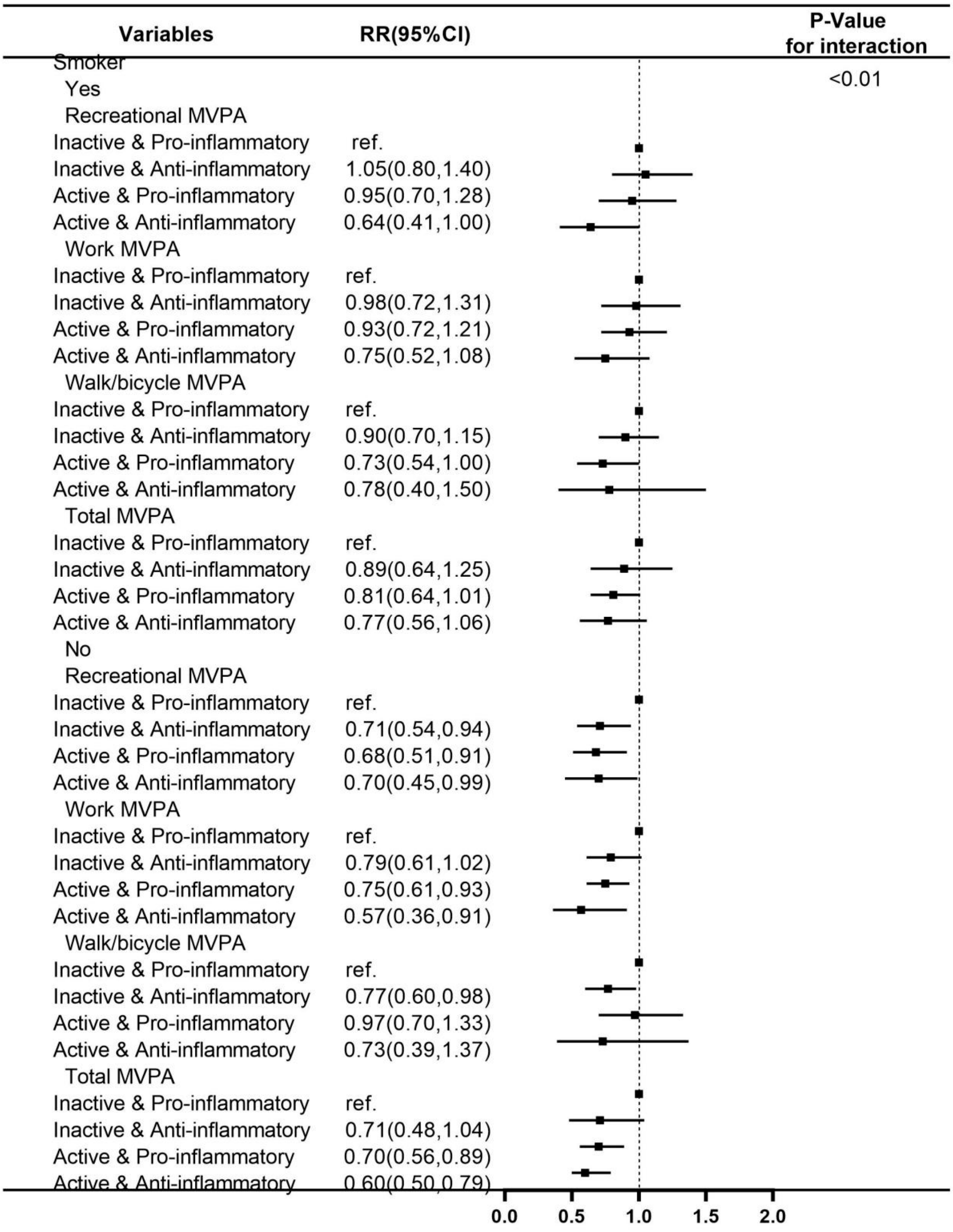

Figure S4. Subgroup and interaction analyses of associations between different domains of PA based on smoking status in combination with inflammatory diet and T2DM.

Variables	RR(95%CI)	P-Value for interaction
Alcohol		
Yes		<0.01
Recreational MVPA		
Inactive & Pro-inflammatory	ref.	
Inactive & Anti-inflammatory	0.88(0.64,1.20)	
Active & Pro-inflammatory	0.76(0.54,1.06)	
Active & Anti-inflammatory	0.64(0.44,0.93)	
Work MVPA		
Inactive & Pro-inflammatory	ref.	
Inactive & Anti-inflammatory	0.87(0.63,1.19)	
Active & Pro-inflammatory	0.75(0.53,1.06)	
Active & Anti-inflammatory	0.61(0.42,0.89)	
Walk/bicycle MVPA		
Inactive & Pro-inflammatory	ref.	
Inactive & Anti-inflammatory	0.83(0.64,1.08)	
Active & Pro-inflammatory	0.76(0.51,1.15)	
Active & Anti-inflammatory	0.72(0.39,1.33)	
Total MVPA		
Inactive & Pro-inflammatory	ref.	
Inactive & Anti-inflammatory	0.67(0.44,1.01)	
Active & Pro-inflammatory	0.62(0.47,0.82)	
Active & Anti-inflammatory	0.63(0.48,0.83)	
No		
Recreational MVPA		
Inactive & Pro-inflammatory	ref.	
Inactive & Anti-inflammatory	0.71(0.46,1.08)	
Active & Pro-inflammatory	0.72(0.48,1.09)	
Active & Anti-inflammatory	0.46(0.19,1.09)	
Work MVPA		
Inactive & Pro-inflammatory	ref.	
Inactive & Anti-inflammatory	0.73(0.48,1.12)	
Active & Pro-inflammatory	0.87(0.57,1.32)	
Active & Anti-inflammatory	0.49(0.22,1.09)	
Walk/bicycle MVPA		
Inactive & Pro-inflammatory	ref.	
Inactive & Anti-inflammatory	0.67(0.43,1.02)	
Active & Pro-inflammatory	0.90(0.57,1.40)	
Active & Anti-inflammatory	0.62(0.19,2.03)	
Total MVPA		
Inactive & Pro-inflammatory	ref.	
Inactive & Anti-inflammatory	0.79(0.47,1.33)	
Active & Pro-inflammatory	0.77(0.55,1.08)	
Active & Anti-inflammatory	0.46(0.25,0.85)	

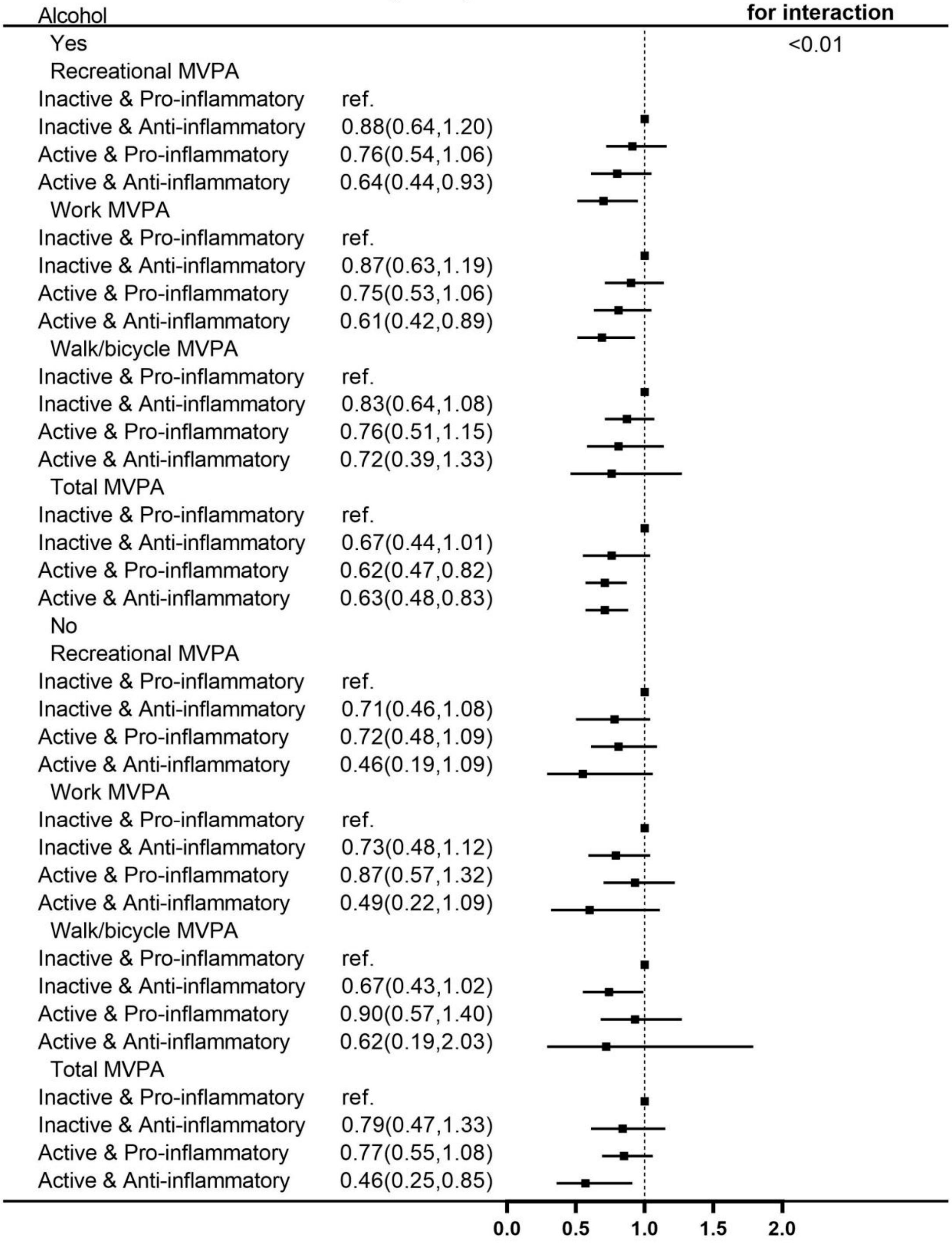

Figure S5. Subgroup and interaction analyses of the association between different domains of PA based on drinking status in combination with inflammatory diet and T2DM.

Variables	RR(95%CI)	P-Value for interaction
BMI		
<25		<0.01
Recreational MVPA		
Inactive & Pro-inflammatory	ref.	
Inactive & Anti-inflammatory	0.79(0.46,1.36)	
Active & Pro-inflammatory	0.72(0.37,1.42)	
Active & Anti-inflammatory	0.66(0.26,1.68)	
Work MVPA		
Inactive & Pro-inflammatory	ref.	
Inactive & Anti-inflammatory	0.83(0.43,1.62)	
Active & Pro-inflammatory	0.72(0.37,1.42)	
Active & Anti-inflammatory	0.56(0.23,1.36)	
Walk/bicycle MVPA		
Inactive & Pro-inflammatory	ref.	
Inactive & Anti-inflammatory	0.80(0.43,1.52)	
Active & Pro-inflammatory	0.67(0.34,1.32)	
Active & Anti-inflammatory	0.60(0.22,1.64)	
Total MVPA		
Inactive & Pro-inflammatory	ref.	
Inactive & Anti-inflammatory	0.54(0.28,1.06)	
Active & Pro-inflammatory	0.67(0.40,1.10)	
Active & Anti-inflammatory	0.67(0.35,1.27)	
>=25&<30		
Recreational MVPA		
Inactive & Pro-inflammatory	ref.	
Inactive & Anti-inflammatory	1.26(0.83,1,93)	
Active & Pro-inflammatory	1.05(0.67,1.65)	
Active & Anti-inflammatory	1.13(0.75,1.69)	
Work MVPA		
Inactive & Pro-inflammatory	ref.	
Inactive & Anti-inflammatory	1.47(1.10,1.99)	
Active & Pro-inflammatory	1.24(0.92,1.67)	
Active & Anti-inflammatory	0.99(0.59,1.80)	
Walk/bicycle MVPA		
Inactive & Pro-inflammatory	ref.	
Inactive & Anti-inflammatory	1.21(0.89,1.65)	
Active & Pro-inflammatory	0.99(0.58,1.70)	
Active & Anti-inflammatory	1.02(0.46,2.24)	
Total MVPA		
Inactive & Pro-inflammatory	ref.	
Inactive & Anti-inflammatory	1.36(0.83,2.22)	
Active & Pro-inflammatory	1.01(0.73,1.39)	
Active & Anti-inflammatory	1.12(0.75,1.69)	
>=30		
Recreational MVPA		
Inactive & Pro-inflammatory	ref.	
Inactive & Anti-inflammatory	0.77(0.62,0.95)	
Active & Pro-inflammatory	0.76(0.60,0.96)	
Active & Anti-inflammatory	0.43(0.27,0.67)	
Work MVPA		
Inactive & Pro-inflammatory	ref.	
Inactive & Anti-inflammatory	0.68(0.54,0.86)	
Active & Pro-inflammatory	0.76(0.62,0.94)	
Active & Anti-inflammatory	0.57(0.39,0.81)	
Walk/bicycle MVPA		
Inactive & Pro-inflammatory	ref.	
Inactive & Anti-inflammatory	0.69(0.56,0.84)	
Active & Pro-inflammatory	0.86(0.64,1.14)	
Active & Anti-inflammatory	0.70(0.43,1.31)	
Total MVPA		
Inactive & Pro-inflammatory	ref.	
Inactive & Anti-inflammatory	0.71(0.53,0.95)	
Active & Pro-inflammatory	0.71(0.60,0.85)	
Active & Anti-inflammatory	0.50(0.37,0.68)	

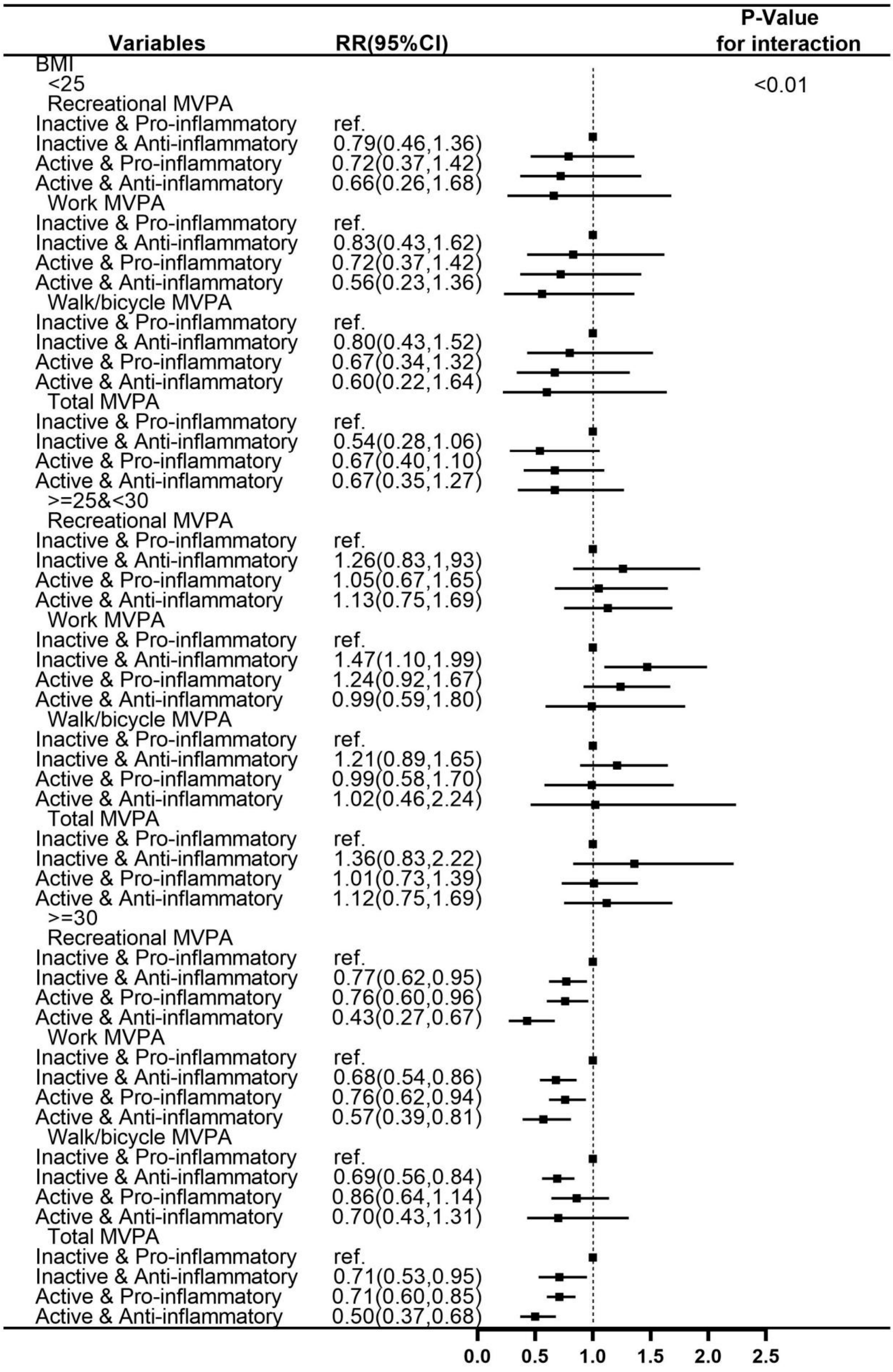

Figure S6. Subgroup and interaction analyses of the association between different domains of PA based on BMI in combination with inflammatory diet and T2DM.

Variables	RR(95%CI)	P-Value for interaction
Model 3		<0.01
Recreational MVPA		
Inactive & Pro-inflammatory	ref.	
Inactive & Anti-inflammatory	0.87(0.72,1.05)	
Active & Pro-inflammatory	0.81(0.67,0.99)	
Active & Anti-inflammatory	0.66(0.49,0.90)	
Work MVPA		
Inactive & Pro-inflammatory	ref.	
Inactive & Anti-inflammatory	0.87(0.72,1.05)	
Active & Pro-inflammatory	0.84(0.70,1.01)	
Active & Anti-inflammatory	0.66(0.49,0.88)	
Walk/bicycle MVPA		
Inactive & Pro-inflammatory	ref.	
Inactive & Anti-inflammatory	0.83(0.70,0.98)	
Active & Pro-inflammatory	0.86(0.70,1.04)	
Active & Anti-inflammatory	0.75(0.48,1.18)	
Total MVPA		
Inactive & Pro-inflammatory	ref.	
Inactive & Anti-inflammatory	0.79(0.61,1.01)	
Active & Pro-inflammatory	0.77(0.65,0.87)	
Active & Anti-inflammatory	0.68(0.55,0.83)	

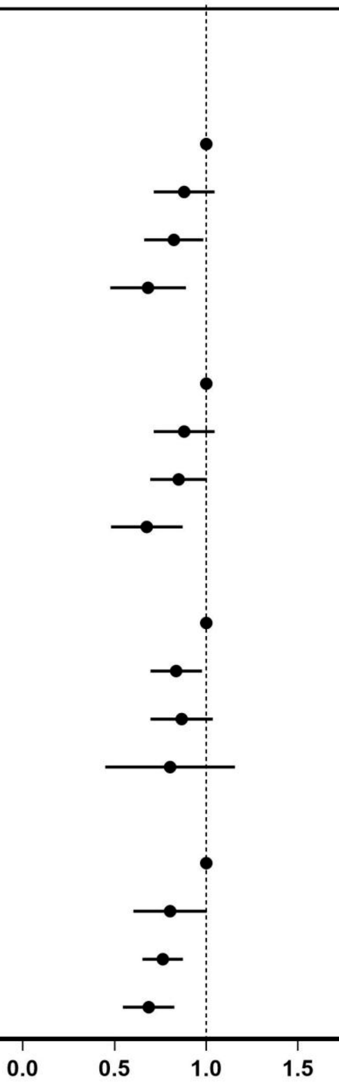

Figure S7. Forest plot of the relationship between physical activity and pro-inflammatory diet with diabetes based on Model 3.
